# The Trimodality PET/CT–MRI (3.0 T) System with a Dedicated Shuttle Detects Local Recurrence in a Case Classified as Indeterminate by Means of PSMA PET/CT Alone at Prostatic Fossa

**DOI:** 10.1055/s-0045-1812055

**Published:** 2025-10-04

**Authors:** Gerardo Gabriel dos Santos Loureiro, Juan Pablo Gambini González, Martin Rodriguez Parodi, Omar Alonso Núñez

**Affiliations:** 1 Uruguayan Centre of Molecular Imaging, Montevideo, Uruguay; 2 Nuclear Medicine and Molecular Imaging Centre, Hospital de Clínicas, Universidad de la República, Montevideo, Uruguay

**Keywords:** PET/CT–MRI, prostate cancer, ^68^ Ga-PSMA-11, biochemical recurrence

## Abstract

In the following case study, we evaluate a prostate cancer patient with persistent biochemical relapse and negative conventional imaging. Following interdisciplinary team discussion, it is decided to assess the patient by a ^11^ C-Choline and ^68^ Ga-PSMA positron emission tomography/computed tomography (PET/CT) scan. Notably, doubtful findings due to physiological urinary activity in the ^68^ Ga-PSMA-11 PET/CT scan were clarified with PET/CT–magnetic resonance imaging (MRI) images. The added value of the trimodality PET/CT–MRI system was very useful in detecting recurrence in a case classified as indeterminate by means of PET/CT alone at prostatic fossa.

## Introduction

The radiolabeled PSMA positron emission tomography/computed tomography (PET/CT) has proven to be a highly accurate method to detect prostate cancer (PCa), especially in the biochemical recurrence (BCR) scenario after definitive intended curative therapy.

Also, the contribution of magnetic resonance imaging (MRI) in hybrid environments (PET/CT–MRI or PET/MRI) has the potential to increase the diagnostic accuracy of ^68^ Ga-PSMA-11 PET/CT scanning.

## Case Report

This is a 60-year-old patient with PCa (pT2c Gleason 6; 3 + 3) with a baseline prostate-specific antigen (PSA) of 7.96 ng/mL at diagnosis, treated with surgery on January 31, 2013, and with no further treatment (no radiotherapy or hormonotherapy). He presented early BCR (less than 1 year), with no lesions detected by ^11^ C-Choline PET/CT (2014) or conventional imaging.

In 2016, with a PSA level 8.4 ng/mL and PSA doubling time of 7 months, the patient underwent both an ^11^ C-Choline and ^68^ Ga-PSMA-11 64-slice PET/CT scanning with time-of-flight correction, within a time window of 1 to 2 weeks, after the injection of 6.0 MBq/kg or 2.0 MBq/kg, respectively.

**Fig. 1:**
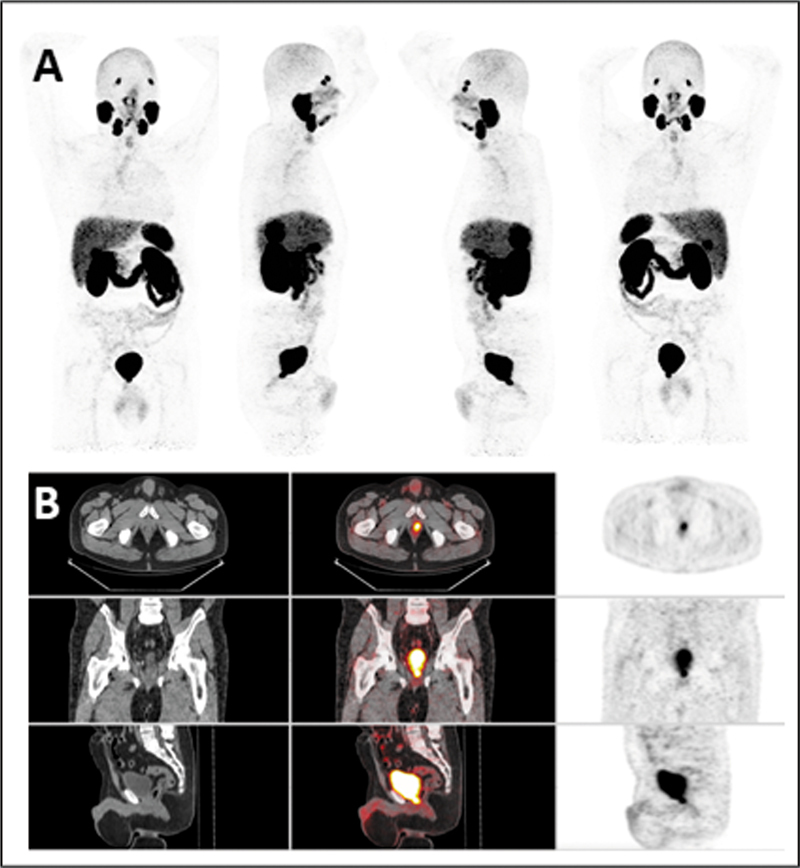
MIP ^68^ Ga-PSMA-11 images (A) of a 60-year-old patient with PCa treated with surgery with elements of biochemical relapse (PSA level 8,4 ng/mL) and negative conventional images. ^68^ Ga-PSMA-11 hybrid images show intense physiological tracer activity in projection to the prostate urethra (bladder infundibulum). PCa, prostate cancer; PSA, prostate-specific antigen.

^11^ C-Choline PET/CT was negative. Conversely, ^68^ Ga-PSMA-11 PET/CT showed intense physiological tracer activity in projection to the prostate urethra (bladder infundibulum) that could obscure the presence of tumor recurrence ( Fig. 1 ).

As a result, additionally, to the ^68^ Ga-PSMA-11 scan, we used a PET/CT–MRI (3.0 T) system with a dedicated shuttle, acquiring MRI images of the pelvis.

**Fig. 2:**
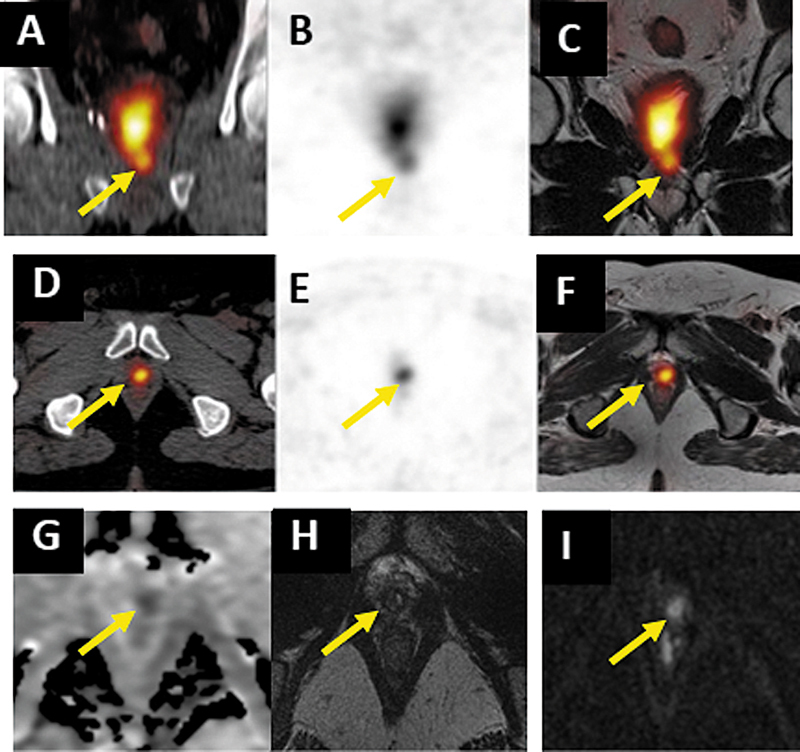
( **A** ) and ( **D** ) PET/CT ^68^ Ga-PSMA-11, ( **B** ) and ( **E** ) PET ^68^ Ga-PSMA-11, ( **C** ) and ( **F** ) PET/MR fusion, axial FSE T2 HD, coronal and axial images, respectively, showing intense and focal uptake of the ^68^ Ga-PSMA-11. No evidence of fluid, which distances the proposal of urine collection. ( **G** ) ADC, ( **H** ) FSE T2 HD, and ( **I** ) DWI, showing a local relapse lesion with restriction that was hidden by physiological tracer bladder activity. The hypointense lesion involves the infundibulum in the left posterolateral prostate region. Sequence and acquisition parameters: RM GE 3T. FSE T2 HD: TE150, reconstruction high. FSE 224. Frequency 352. Nex 1.50. Thickness 3 mm. Auto TR 4294 DWI-ADC focus axial FOV FREC 15, thickness 3 mm. Frequency direction R-L, auto TR 4877. *b* -Values 2: 50–1000. Synthetic *b* -value: 500–15,000–2,000. DWI, diffusion-weighted imaging; MR, magnetic resonance; PET/CT,

Although the ^68^ Ga-PSMA-11 PET/MRI images showed no abnormal pelvic findings, the diffusion-weighted imaging and high-resolution T2 magnetic resonance (MR) scans showed a local relapse lesion with restriction that was hidden by physiological tracer bladder activity. The hypointense lesion involved the infundibulum in the left posterolateral prostate region ( Fig. 2 ).

Radiotherapy was performed on this region with lesion stability until 2024.

**Fig. 3:**
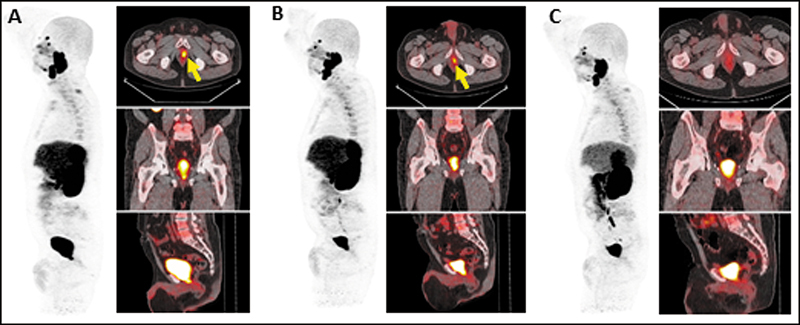
Sequential ^18^ F-AlF-PSMA PET/CT scans (2016 and 2021, **A** and **B** , respectively) showing persistence of known area of hyperuptake in the left posterolateral bladder neck compatible with tumor activity (yellow arrows). After radiotherapy and hormonotherapy treatment, the last ^18^ F-AlF-PSMA PET/CT performed in December 2014 was negative.

Evaluated by oncological urology, he was started on hormone therapy. PSA drops from 8 to 1.5 and then 0.84 ng/mL. The last ^18^ F-AlF-PSMA PET/CT was negative ( Fig. 3 ).

## Discussion

PCa is the second most common malignant tumor in men and one of the most common malignancies in the world. 1

One of the key clinical issues of this disease is the diagnosis of PCa recurrence in patients with BCR. 2

Radiolabeled choline PET/CT has been widely used but has a relatively low sensitivity at low PSA levels. 3

The usefulness of PET/CT with ^68^ GA-PSMA-11 in this scenario has been amply demonstrated in several clinical studies, which have confirmed a preferential uptake by PCa and their metastases, especially at low levels of PSA (<0.5 ng/mL) with the potential of changing clinical management. 4

Nevertheless, ^68^ Ga-PSMA-11 ligands are excreted foremost via the urinary system and collected in the bladder. Thus, some small local recurrences might be missed, even with the use of diuretics (forced diuresis) or delayed images. 5 The protocol used in our center for reducing physiological activity in the bladder includes patient hydration and the acquisition of delayed images.

According to Afshar-Oromieh et al., the introduction of novel molecular imaging techniques, such as MRI, in recent years may offer clinicians valuable information that can influence the management of PCa patients. 6

The detection of local and regional recurrence after treatment in PCa patients has been demonstrated to be highly sensitive and specific using multiparametric MRI. 7 The recent application of PET/MRI employing with ^68^ Ga-PSMA-11 shows encouraging outcomes. This combination provides excellent morphological detail, multiparameter functional information, and molecular data.

Within this framework, the trimodality PET/CT–MRI system facilitates the transport of the patient via a specialized shuttle from one imaging modality to another, ensuring that the patient's position remains unchanged. This novel sequential imaging technique could lead to a significant improvement in the detection of PCa. 8

Afshar-Oromieh et al. 6 and Eiber et al. 9 investigated the feasibility of PET/MRI hybrid system using ^68^ Ga-PSMA-11. Their research revealed that PCa was detected more easily and accurately with the ^68^ Ga-PSMA-11 PET/MRI hybrid system compared with PET/CT, while also minimizing radiation exposure. As a result, this innovative technique could clarify unclear PET/CT results, regardless of PSA levels.

The hybrid PET/CT–MRI (3.0 T) system represents a viable imaging technique that potentially adds useful pertinent information, enhancing diagnostic precision. The benefits of the trimodality PET/CT–MRI system include a more accurate attenuation correction, reliable PET quantification, superior soft tissue contrast and a higher imaging flexibility that improves diagnostic precision for PCa. 7

The sequential acquisition of the techniques minimizes issues related to misalignment and patient repositioning.

A key discovery in this case report is that MRI is highly useful in detecting recurrence in cases classified as indeterminate at prostatic fossa assessed solely through PET/CT, particularly in patients with local recurrences observed near the bladder as described in the literature. 9

In such patients, PET/CT probes incapable of detecting a local recurrence due to physiological tracer activity in the bladder, while MR clearly indicates pathology.

In cases where PET/CT fails to reveal a local relapse despite strong concern about its presence, it would be advisable to complete the study with an MRI of the region, considering the superior efficacy of this technique in these circumstances.

## Conclusion

The trimodality PET/CT–MRI system demonstrates superior diagnostic performance in detecting local recurrence obscured by urinary tracer activity and should be considered in equivocal PSMA PET/CT cases.
